# The Effect of Diiodooctane on the Charge Carrier Generation in Organic Solar Cells Based on the Copolymer PBDTTT-C

**DOI:** 10.1038/srep08286

**Published:** 2015-02-06

**Authors:** Andreas Zusan, Björn Gieseking, Mario Zerson, Vladimir Dyakonov, Robert Magerle, Carsten Deibel

**Affiliations:** 1 Experimental Physics VI, Julius-Maximilians-University of Würzburg, 97074 Würzburg, Germany; 2 Fakultät für Naturwissenschaften, Technische Universität Chemnitz, 09126 Chemnitz, Germany; 3 Bavarian Center for Applied Energy Research e.V. (ZAE Bayern), 97074 Würzburg, Germany

## Abstract

Microstructural changes and the understanding of their effect on photocurrent generation are key aspects for improving the efficiency of organic photovoltaic devices. We analyze the impact of a systematically increased amount of the solvent additive diiodooctane (DIO) on the morphology of PBDTTT-C:PC_71_BM blends and related changes in free carrier formation and recombination by combining surface imaging, photophysical and charge extraction techniques. We identify agglomerates visible in AFM images of the 0% DIO blend as PC_71_BM domains embedded in an intermixed matrix phase. With the addition of DIO, a decrease in the size of fullerene domains along with a demixing of the matrix phase appears for 0.6% and 1% DIO. Surprisingly, transient absorption spectroscopy reveals an efficient photogeneration already for the smallest amount of DIO, although the largest efficiency is found for 3% DIO. It is ascribed to a fine-tuning of the blend morphology in terms of the formation of interpenetrating donor and acceptor phases minimizing geminate and nongeminate recombination as indicated by charge extraction experiments. An increase in the DIO content to 10% adversely affects the photovoltaic performance, most probably due to an inefficient free carrier formation and trapping in a less interconnected donor-acceptor network.

The high photovoltaic performance of state-of-the-art organic bulk heterojunction (BHJ) solar cells has been mainly driven by the development of novel copolymer donor materials^1^. A further increase in the power conversion efficiency (PCE) requires a detailed control of the active layer morphology, which is critical to both the conversion of photoexcitations into free charge carriers as well as their extraction^2^,^3^,^4^,^5^,^6^,^7^. While the microstructure of poly(3-hexylthiophene-2,5-diyl) (P3HT) blended with the fullerene acceptor [6,6]-phenyl-C61-butyric acid methyl ester (PC_61_BM) can be tuned by thermal annealing^8^, the morphology of active layers based on low-bandgap copolymers is usually optimized through the incorporation of small amounts of solvent additives, e.g., diiodooctane (DIO), in the blend solution from which the BHJ layers are cast^9^,^10^,^11^,^12^,^13^. Typically, these additives act as selective solvent of the fullerene molecules in the processing solution and thus affect their separation during formation of the active layer. As a result, the morphology of donor (D) and acceptor (A) phases is significantly altered. Using DIO, the PCE of devices based on the high performance copolymer poly[(4,8-bis-(2-ethylhexyloxy)-benzo(1,2-b:4,5-b′)dithiophene)-2,6-diyl-alt-(4-(2-ethylhexyl)-3-fluorothieno[3,4-b]thiophene-)-2-carboxylate-2-6-diyl)] (PTB7) blended with [6,6]-phenyl-C71-butyric acid methyl ester (PC_71_BM) can be increased up to 9.2%^14^,^15^,^16^. On the one hand, this is explained by a reduced size of fullerene aggregates resulting in an overall enhanced D-A intermixing^17^,^18^. On the other hand, Collins et al. and Hedley et al. suggest that DIO reduces fullerene domain size, with only minor effects on domain composition and crystallinity^19^, and supports the formation of elongated fiber-like polymer-rich and fullerene-rich domains in the optimized blend^20^, respectively. While all these studies address the comparison of the active layer without additive to the one with the optimum amount of DIO, little is known about the direct correlation between the additive content and the effects of morphological changes on free charge carrier formation and recombination losses, leading to the distinct maximum of the PCE for a certain additive concentration.

In this work we report on the effect of a systematically increased amount of DIO on the morphological, photophysical and electrical properties of solar cells composed of poly[(4,8-bis-(2-ethylhexyloxy)-benzo(1,2-b:4,5-b′)dithiophene)-2,6-diyl-alt-(4-(2-ethylhexanoyl)-thieno[3,4-b]thiophene-)-2-6-diyl)] (PBDTTT-C) and PC_71_BM. PBDTTT-C (Fig. 1a) is a member of the family of benzodithiophene copolymers with a chemical structure similar to PTB7, yielding a PCE up to 7.4%^21^,^22^,^23^. We apply intermittent contact mode atomic force microscopy (IC-AFM), picosecond time-resolved photoluminescence (PL) and transient absorption (TA) spectroscopy to study the impact of DIO on the topography, the nanomorphology and the efficiency of fast photogeneration. Complementary time delayed collection field (TDCF)^24^ and open circuit corrected transient charge extraction (OTRACE)^25^ measurements yield information about the field dependence of free charge carrier formation and nongeminate recombination dynamics. We find a morphology of the sample processed without DIO characterized by large acceptor agglomerates embedded within an intermixed D-A phase, resulting in a relatively poor PCE. With the addition of DIO, our measurements show a decrease in the size of PC_71_BM domains, setting in for an amount of 0.6% DIO, as well as the demixing of the matrix phase starting at a fraction of 1% DIO and leading to the formation of pure donor regions. The altered microstructure results in a highly efficient photogeneration already for the smallest amount of DIO. However, only the formation of an interpenetrating D-A network facilitates the collection and extraction of free charge carriers yielding a maximum PCE for the 3% DIO blend. The further increase in the DIO content up to 10% reduces the PCE, which appears to result from once again less interconnected pure domains.

## Results and Discussion

### Current–voltage characteristics

**Figure 1:**
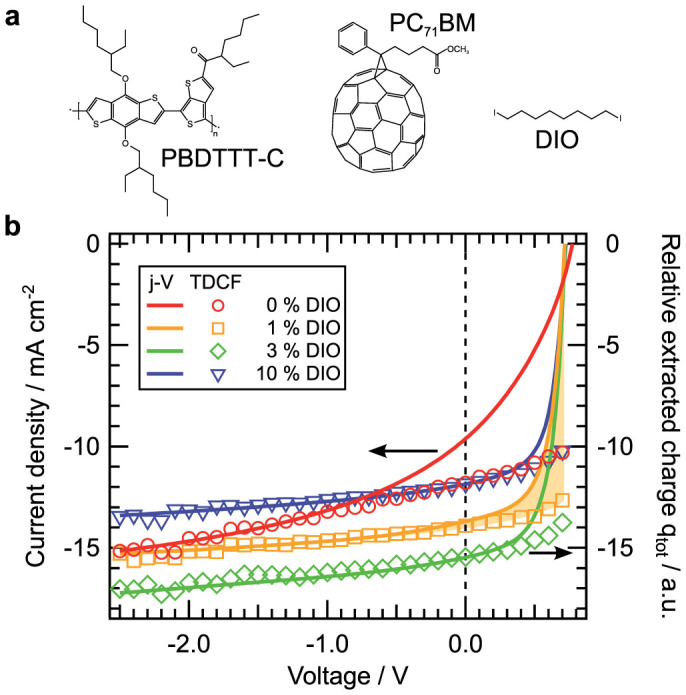
Photovoltaic characterization and field dependent photogeneration. (a) Molecular structure of PBDTTT-C, PC_71_BM and DIO. (b) *j*-*V* characteristics (left axis) and relative extracted charge carrier density *q_tot_* derived from TDCF (right axis) of PBDTTT-C:PC_71_BM solar cells with varying DIO content. The illumination intensity was set to 1 sun. *q_tot_* was normalized to the current density at −2.5 V. The difference between *j(V)* and *q_tot_*corresponds to nongeminate recombination losses as indicated by the shaded area using the example of 1% DIO.

The current-voltage (*j*-*V*) characteristics of the PBDTTT-C:PC_71_BM devices with increasing DIO content (0%, 1%, 3%, and 10%) are shown in Fig. 1b. The devices were selected from a series of more than ten solar cells, each. A more detailed analysis of the *j*-*V* characteristics can be found in Supplementary Fig. S1 and Table S1. A maximum PCE of 6.9% was found for the device with 3% DIO. It results from an increase of 61% and 74% in the short circuit current density (*j_sc_*) and fill factor (FF), respectively, compared to the blend without additive, overcompensating the slight decrease of about 7% in the open circuit voltage (*V_oc_*). The most substantial boost in device performance, however, already results from the addition of 0.6% and 1% DIO. The PCE of the device with 10% DIO remains at a high level, although *j_sc_* is reduced by roughly a quarter compared to the best device with 3% DIO. In addition to the *j*-*V* characteristics, Fig. 1b depicts the relative extracted charge carrier density derived from TDCF measurements (right axis), which will be discussed below.

### Surface morphology

**Figure 2:**
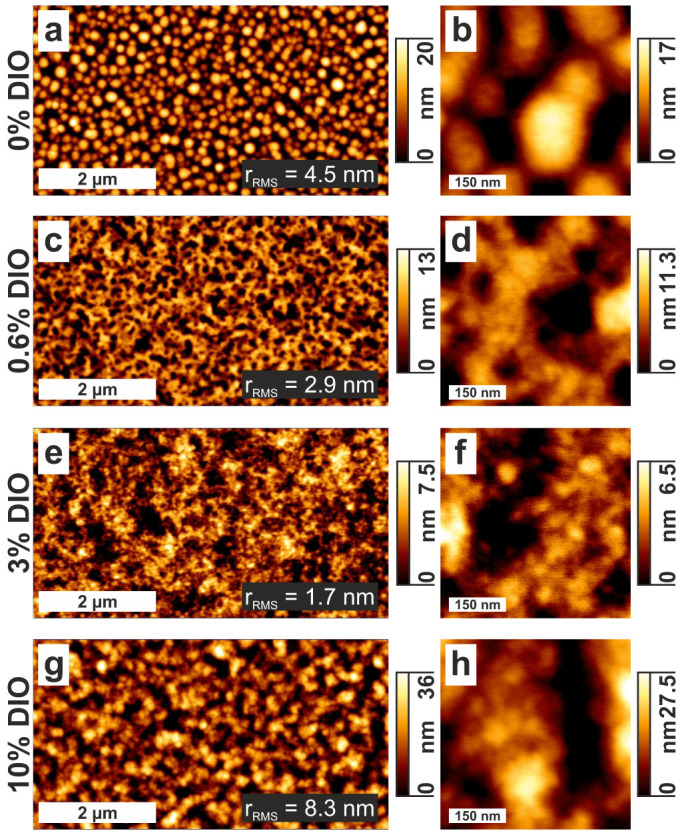
Surface morphology of the studied blends. Large-area (left) and high resolution (right) IC-AFM height images of thin films of PBDTTT-C:PC_71_BM prepared with different amounts of DIO.

The surface morphologies of thin films of PBDTTT-C:PC_71_BM blends were studied using IC-AFM. The left column of Fig. 2 shows large-area IC-AFM height images of samples prepared with different amounts of DIO (0%, 0.6%, 3%, and 10%). The sample prepared without DIO (Fig. 2a) shows agglomerates in the form of round domains (flakes) and a root mean square roughness (*r_RMS_*) of 4.5 nm. With the addition of 0.6% DIO, the surface roughness decreases to 2.9 nm and the surface structure resembles the morphology of a collapsed network of about 10 nm large particles. The on average 50 to 200 nm wide and 10 nm deep depressions (Fig. 2c) are attributed to the pores of the network that is formed during spin-casting when the CB evaporates and a gel-phase is formed. Upon evaporation of DIO, this network collapses. With further addition of up to 3% DIO (Fig. 2e) the surface morphology remains the same, but with smaller features (particles and pores) and the lowest *r_RMS_* of 1.7 nm. In contrast to this trend, the sample prepared with 10% DIO (Fig. 2g) displays again larger agglomerates in form of round domains as the sample prepared without DIO and a *r_RMS_* of 8.3 nm. High resolution images of the samples prepared with different amounts of DIO are shown in the right column of Fig. 2. The topography of the sample prepared without DIO clearly shows round domains with an average diameter between 100 and 200 nm. Increasing the amount of DIO up to 3% results in a morphology on the nanometer length scale characterized by the decreasing size of round domains as well as the agglomeration of particles with increasing DIO content.

Similar thin film morphologies and changes upon the addition of DIO were observed by Collins et al. for PTB7:PC_71_BM blends using X-ray scattering techniques^19^. Their results reveal that the large round domains consist of pure PC_71_BM and are embedded in a PTB7-rich matrix with a composition equal to the thermodynamic miscibility. With the addition of 3% DIO, the size of PC_71_BM domains drastically decreases, whereas neither the domain composition nor the crystallinity changes. The surface morphology of PBDTTT-C:PC_71_BM blends can be interpreted along the same lines: The on-average 100 to 200 nm large, round domains formed in the sample prepared without DIO (Fig. 2b) most probably consist of PC_71_BM and are embedded in a PBDTTT-C-rich matrix. With the addition of up to 3% DIO these domains get smaller, leading to a drastically increased interfacial area between the donor and the acceptor material and to a morphology that is ideal for free charge carrier generation and collection. However, it must be emphasized that AFM is a surface sensitive technique allowing only limited conclusions regarding to the bulk morphology. In contrast to the clear variations of the surface morphologies visible in Fig. 2, IC-AFM phase images (Supplementary Fig. S2) show a nanoscale structure with no major differences between the samples prepared with different amounts of DIO. One reason for this behavior might be the presence of a PBDTTT-C-rich skin layer on top of the sample, forming a nanoscale, near-surface morphology that does not depend on the amount of DIO. This structural model resembles the nanoscale morphology observed in spin-cast films of blends of a PPV derivate with PC_61_BM^26^. Above the solubility limit of PC_61_BM in the PPV derivate, PC_61_BM domains are formed, that are completely covered by a PPV-rich matrix phase. The existence of a similar top skin was recently reported for PTB7:PC_71_BM blend films^20^. It must be emphasized that despite the presence of a thin, polymer-rich top skin, round domains visible in AFM can be related to agglomerated PC_71_BM below that layer.

### Nanomorphology and fast photogeneration

**Figure 3:**
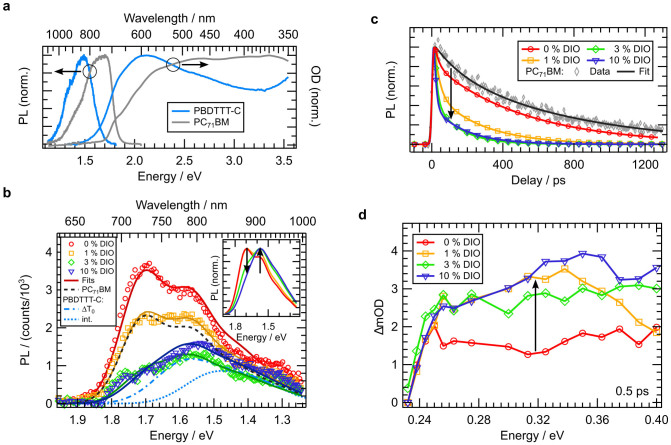
Photophysical properties. (a) Absorbance and PL spectra of neat films of the donor and acceptor materials. (b) Time-integrated PL spectra of the blend films (symbols) and corresponding fits according to Equation (1) (solid lines). For comparison also the time-integrated PL spectra of the fullerene film (dashed line) as well as the initial polymer spectrum (dash-dotted line) are shown. Both curves were scaled with the fit amplitudes *A_A_* and *A_D_* of the 1% and 10% DIO sample (Supplementary Table S2), respectively. The dotted line corresponds to the integral polymer emission. The inset illustrates the relative spectral change of the fitted spectra. (c) Fits of PL transients taken at 1.74 eV reflecting the decay dynamics of excitons generated within the fullerene domains. For comparison also the PL decay of a neat fullerene film is shown. (d) Initial (0.5 ps) TA spectra probed in the 0.24 to 0.4 eV region. Already the use of 1% DIO results in an enhanced initial generation of bound and free charge carriers (arrow) staying on a similarly high level for the 3% and 10% film.

To gain deeper insight into the effect of DIO on the bulk photogeneration, photophysical measurements were performed. As a first step, the singlet exciton dynamics of the respective blends was analyzed using time-resolved PL measurements. Since the films were excited at 3.1 eV, excitons were created in both donor and acceptor (Fig. 3a), resulting in emission spectra that show contributions from both, PBDTTT-C and PC_71_BM. Due to the quenching at the D-A interfaces the donor emission can only be observed around zero delay without exhibiting the prominent dynamic redshift induced by exciton diffusion as it was observed for neat polymer films (data not shown)^27^. This suppression of the redshift indicates a fast and efficient polymer singlet exciton quenching by dissociation at the D-A interfaces. Hence, in order to correctly describe the blend emission, the separately recorded initial donor PL spectrum has to be employed while the acceptor contribution can be modeled using the time-integrated emission spectrum.

The time-integrated PL spectra (0 ps to 1.5 ns) for films of blend compositions with 0%, 1%, 3%, and 10% DIO are shown in Fig. 3b. The shape of the PL spectra can be qualitatively reproduced by a superposition of both individual spectra:
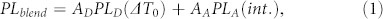
with *PL_D_(ΔT_0_)* being the initial, zero delay donor PL (Fig. 3b, dash-dotted line) and *PL_A_(int.)* the time-integrated acceptor PL spectrum (Fig. 3b, dashed line), respectively. *A_D,A_* are the corresponding amplitudes (fitting parameters can be found in Supplementary Table S2). The blend spectra do not exhibit significant contributions of charge transfer (CT) state emission within the analyzed spectral range.

The PL spectrum of the blend without DIO can be completely described by the emission from the fullerene. This means that a significant amount of excitons is generated within acceptor domains with a size exceeding the typical exciton diffusion length *d_e_* of about 5 to 10 nm as reported for organic semiconductors^20^,^28^,^29^,^30^,^31^,^32^. Thus, a radiative decay occurs before the excitons can reach the D-A interface. The absence of donor emission points towards the presence of an additional well intermixed D-A phase in the active layer bulk, showing a length scale of D and A domains on the order of or smaller than *d_e_*. The observation confirms the identification of large agglomerates observed in the respective AFM image with fullerene domains embedded in an intermixed D-A matrix at the film surface. Increasing the DIO content up to 3% results in a gradual decrease in blend PL. The overall reduction stems from a substantially reduced emission from PC_71_BM but an increased contribution of donor PL (Supplementary Table S2). The former is again consistent with the AFM data indicating an enhanced intermixing and the formation of an acceptor network. For the 10% blend, a slight increase in the blend PL can be observed as compared to 3% DIO resulting from a gradual decrease in acceptor emission and an increase in donor emission (Supplementary Table S2). As a consequence, the shape of the blend spectrum changes from being acceptor dominated to being donor dominated upon the systematic increase of DIO from 1% to 10% (Fig. 3b, inset). The change in the time-integrated PL spectra upon increasing the DIO content are related to both a reduced size of fullerene domains as well as the formation of additional pure donor regions within the intermixed phase. The observation of an enhanced quenching of the acceptor emission is only possible, if excitons generated within fullerene domains reach the D-A interface, requiring a size of acceptor phases on the order of the aforementioned *d_e_*. By analogy, the strongly increasing polymer emission implies a length scale of the donor domains larger than the exciton diffusion length. Thus, the alteration of the blend morphology is assumed to appear on the 10 nm length scale. It is important to mention that an intimate D-A intermixing on the molecular scale is expected to result in a more pronounced PL quenching.

To verify our interpretation of the influence of DIO content on the sample morphology, we compared the PL decay dynamics at 1.74 eV for the different blends (Fig. 3c). Without DIO, they mainly correspond to the decay dynamics of excitons generated on fullerenes, as seen in comparison to the PL decay of a neat PC_71_BM film: In the absence of PBDTTT-C, the effective radiative lifetime *τ_2_* of fullerene singlet excitons is approximately 700 ps (fitting parameters can be found in Supplementary Table S2). In the blend *τ_2_* is reduced and a second fast picosecond channel *τ_1_* is introduced. The time constant of this fast channel is close to the temporal resolution of our measurements and probably results from the dissociation of excitons at the D-A interface. Adding DIO and increasing its content leads to a strongly enhanced relative contribution of *τ_1_*, which can be explained with a reduced average acceptor domain size allowing more singlet excitons to reach a heterointerface during their migration. This observation is in agreement with the reduction of the average size of fullerene domains indicated in the AFM images. Furthermore, the PL transient for the 10% DIO film shows a strongly enhanced contribution of *τ_1_* and slight increase in *τ_2_* relative to the 3% DIO blend.

Both the integral PL and the decay dynamics indicate a reduction of the size of fullerene domains with increasing DIO fraction. This implies a finer dispersion of donor and acceptor phases, which is expected to significantly enhance the density of initially photogenerated charge carriers due to the increased interfacial area between both phases. To study the charge carrier photogeneration directly, we performed transient absorption spectroscopy. In contrast to the TDCF method both free and bound charge pairs can be detected in TA measurements. The initial rise of the recorded transient signal in the IR region between 0.24 and 0.4 eV is directly proportional to the population of the photogenerated polarons and/or bound polaron pairs (i.e., CT complexes)^33^,^34^,^35^,^36^, without spectrally overlapping intrachain excitations^37^. Figure 3d shows the transient absorption spectra of the four samples at 0.5 ps. We point out that due to an overall reduced transmission of the setup the detectable change in optical density is significantly reduced below 0.25 eV. Comparing the spectra, it becomes obvious that already the addition of 1% DIO results in an increase in the transient absorption by more than a factor of two. As compared to the 1% DIO blend, a higher DIO content of 3% and 10% does not lead to significant changes. The enhanced fast photogeneration already for the use of 1% DIO (Fig. 3d, arrow) can be explained by a significant increase in D-A interfacial area as a result of the presence of smaller fullerene domains, supporting the results of the time-resolved PL measurements. The finding is also in agreement with the increase in PCE of the device with 1% DIO (Fig. 1b and Supplementary Fig. S1). At first glance, no considerable changes of the initial TA signal for the 3% DIO blend disagree with AFM and PL measurements, that indicate smaller PC_71_BM domains and thus an increase in interfacial area when adding 3% of DIO. Based on the enhanced donor PL of the 3% DIO blend (Supplementary Table S2), these observations can be explained by the formation of pure polymer regions within the intermixed D-A phase compensating the positive effect of shrinking acceptor domains. The same reasoning applies for the 10% DIO sample showing both an even more donor dominated PL emission as well as larger agglomerates visible in AFM. It can be speculated that a slightly reduced photogeneration for 3% DIO and an improved photogeneration for 10% DIO relative to 1% result from the interplay of shrinking acceptor domains and the formation of pure polymer regions.

**Figure 4:**
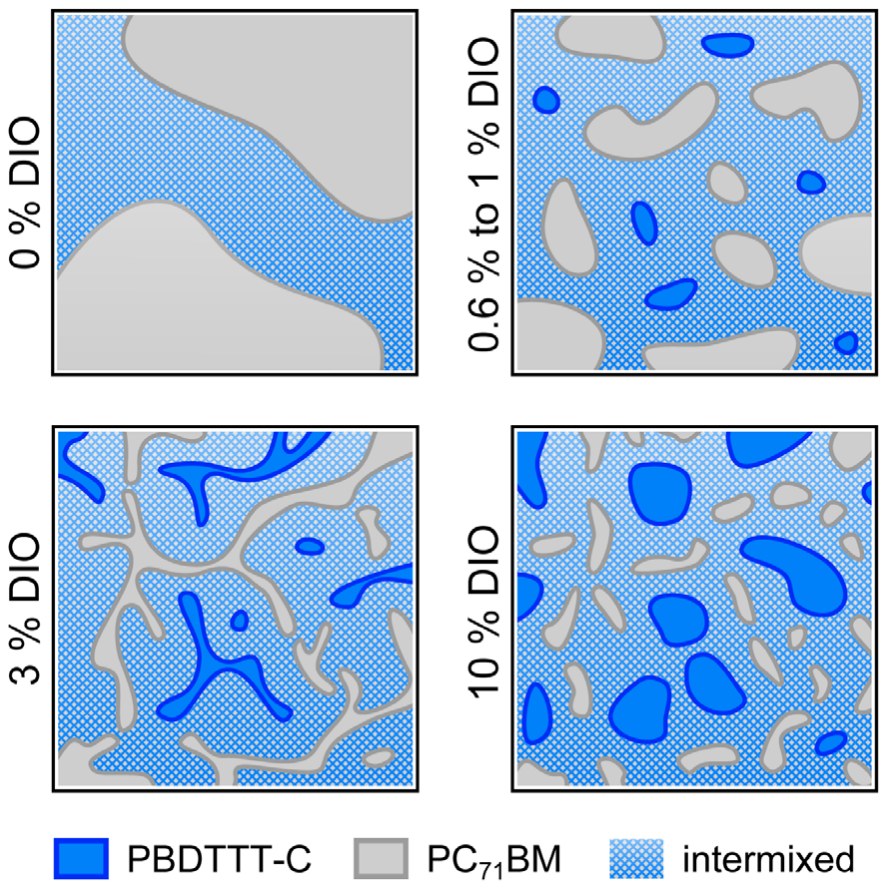
Schematic illustration of the dominant transformations of the blend morphology caused by the use of DIO. The use of small amounts of DIO (0.6% to 1%) leads to the substantial decrease in the size of pure fullerene domains surrounded by an intermixed D-A phase. It is followed by a continuing fine-tuning of the blend morphology. The optimum tradeoff between domain size and linking of pure domains, i.e., photogeneration and collection of free charge carriers, is achieved by the use of 3% DIO yielding a maximum PCE.

Despite a comparable yield of fast photogeneration of the 1%, 3%, and 10% DIO blends, the PCE peaks at 3% DIO (Fig. 1b and Supplementary Fig. S1). In this regard, one has to take a look on the decay dynamics probed by TA first (Supplementary Fig. S3 and Table S2). The transients were found to be independent of the photon energy of the probe beam and do not change significantly upon variation of the DIO content. They can be fitted assuming a biexponential decay yielding a fast and a slow time constant of *τ_1_* ≈ 100 ps and *τ_2_* ≈ 2 ns, respectively. These decay times are characteristic for the decay of bound charge pairs^38^. Hence, it can be assumed that the transient absorption of the 1% and 10% DIO samples is dominated to a greater extent by bound polaron pairs than in the case of 3% DIO. The maximum PCE of the 3% DIO device therefore presumably results rather from a fine-tuning of the blend nanostructure with respect to the formation of percolation paths and an efficient collection of free charge carriers than a profound change of morphology as observed for the addition of small amounts of DIO, i.e., 0.6% and 1% DIO. The results of AFM and photophysical measurements allow to draft the scenario presented in Fig. 4. The drawing should be understood as an illustration of the assumed most significant changes of the blend microstructure upon the addition of DIO, but not as a reproduction of the actual blend morphology. It is important to note that the precise effect of 10% DIO is difficult to establish as it will be discussed below in the context of OTRACE measurements.

### Field dependent photogeneration and nongeminate recombination

To complete the study of the effect of microstructural changes on the photovoltaic performance of PBDTTT-C:PC_71_BM blends, charge extraction measurements were performed. Compared to our results by photophysical techniques, the following experiments require operational devices and thus are sensitive to free, extractable charge carriers. In order to gain information about the yield of free charge carrier formation on the 10 ns scale, pre-bias dependent TDCF measurements were performed. To exclude nongeminate losses, the pulse fluence was adjusted to be in a range showing a photogeneration linearly proportional to the illumination intensity (Supplementary Fig. S4). The extracted charge carrier density *q_tot_* obtained from TDCF transients can be found in Fig. 1b (right axis) and for the full pre-bias range in Supplementary Fig. S5. For all devices, *q_tot_* decreases towards lower internal electric fields, which is commonly linked with a field dependent charge carrier photogeneration via bound CT complexes^39^,^40^,^41^. Regarding the 0% DIO blend, *q_tot_* is reduced by about 65% when going from −4 V to zero internal electric field close to *V_oc_*. The decrease in *q_tot_* in this voltage range is diminished by the addition of DIO, yielding a descent to about 80% (0.6% DIO) and 85% (1% and 3% DIO) of the primary saturation value at a pre-bias close to *V_oc_*, respectively. The further addition of DIO up to 10% DIO results in the reversed trend and an again increased field dependence, with a drop to about 80% of the saturation value at a pre-bias close to *V_oc_*. In addition to explaining the field dependent decay of *q_tot_* with local geminate losses, the extraction of separated charge carriers from pure domains towards percolation paths has to be considered. Recently, Burkhard et al. showed the generation of free charge carriers inside large PC_71_BM domains at an excitation energy of 2.33 eV^42^. They stated that charge carriers overcome recombination by a hole transfer to the polymer, which is favored by an applied external electric field. This field dependent separation is a possible cause for the strong geminate losses in the case of the 0% DIO device. Hence, the reduction of the field dependence of *q_tot_* when using 0.6% DIO might result from a more efficient hole transfer most likely due to shrinking PC_71_BM domains. The observation affirms the findings in the previous paragraph, revealing the same profound change in blend morphology for adding the smallest amount of DIO. With 1% and 3% DIO, TDCF shows a further reduced field dependence indicating the formation of percolation pathways between pure domains as it was recently demonstrated for another material system^43^. The reversed trend of an increased field dependence in the case of 10% DIO thus implies again less interconnected pure domains. The interpretation is consistent with the results shown in the last paragraph suggesting the most efficient collection of free charge carriers in an optimized interpenetrating D-A network for 3% DIO. At first sight, the reduced pre-bias dependence along with a decreasing size of the fullerene domains seems inconsistent with current publications reporting a more efficient charge carrier photogeneration with an increasing size of PCBM domains^44^,^45^,^46^,^47^,^48^,^49^. However, for PBDTTT-C:PC_71_BM, the decrease in the size of fullerene domains happens on a considerably larger length scale, starting at 100 to 200 nm for 0% DIO.

In Fig. 1b, *q_tot_* is normalized to the current density of the investigated devices at −2.5 V and compared to the *j*-*V* characteristics. Since the difference between *q_tot_* and *j(V)* (Fig. 1b, shaded area) corresponds to nongeminate losses, the comparison allows to determine the influence of geminate and nongeminate recombination on device performance^40^,^50^,^51^. The field dependence of *q_tot_* is largest for the 0% DIO device and *j(V)* and TDCF agree only for *V <* −1 V, indicating that the photovoltaic function is affected by both strong geminate and nongeminate losses. By the use of 1% and 3% DIO, the field dependence of *q_tot_* is reduced and a discrepancy between *j(V)* and TDCF can only be found in a small bias range close to *V_oc_*. The observation can be interpreted in terms of a higher yield of free carrier formation in combination with minimized nongeminate losses. In contrast, Hawks et al. identified nongeminate recombination alone as the dominant loss mechanism in PBDTTT-C:PC_71_BM solar cells (3% DIO) verified by *j*-*V* reconstructions assuming a field independent generation rate^52^. It is also striking to see that the recombination dynamics in PBDTTT-C:PC_71_BM clearly differ from closely related PTB7 based blends. Recently, Foster et al. reported on a similarly reduced geminate recombination but enhanced nongeminate losses, i.e., a reduced charge carrier lifetime, for PTB7:PC_61_BM devices optimized through the use of 3% DIO^53^. The comparable result was found by Foertig et al. for PTB7:PC_71_BM devices^54^.

**Figure 5:**
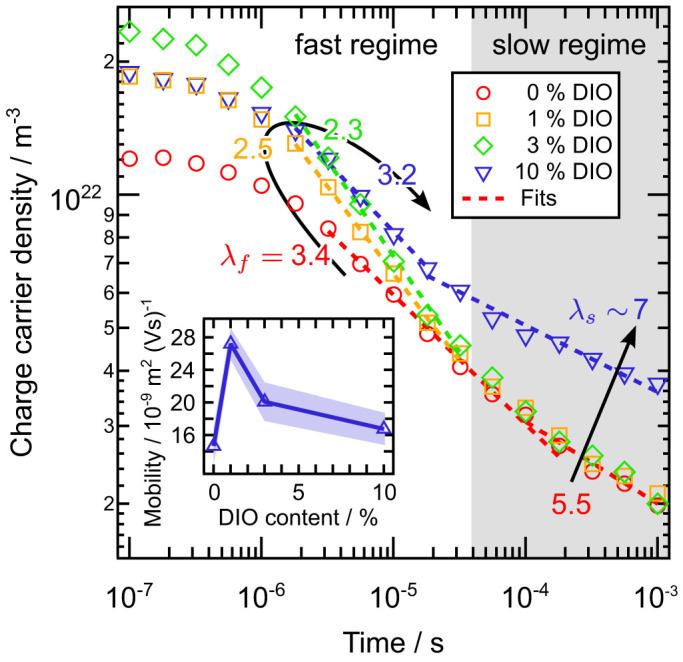
Nongeminate recombination dynamics and charge carrier mobility. Extracted charge carrier density *n* vs. *t_d_* derived from OTRACE measurements under 1 sun illumination intensity on PBDTTT-C:PC_71_BM devices with varying DIO content. The arrows indicate the trend of the inverse slope *λ* derived from a power law fit *n(t) ~ t^−1/λ^* in the fast and slow recombination regime (shaded). The inset shows the charge carrier mobility *μ* vs. DIO content obtained from OTRACE transients. The shaded area indicates the standard deviation.

In the following the charge extraction technique OTRACE is applied to study details of nongeminate recombination dynamics and charge carrier transport. The inset in Fig. 5 presents the effective charge carrier mobility *μ* for various DIO contents derived from the maximum peak position of OTRACE transients obtained under 1 sun illumination intensity^55^. Details about the derivation of *μ* can be found in Supplementary Fig. S7. The devices show values of *μ* on the order of 2**·**10^−8^ m^2^ (Vs)^−1^, which is common for organic solar cells^25^. Surprisingly, the use of DIO leads to an increase in *μ* which might be explained by an improved crystallinity of PBDTTT-C:PC_71_BM blends. However, based on the almost unchanged degree of order in PTB7:PC_71_BM blends without and with DIO^17^,^19^, it seems to be more appropriate to relate this finding to the formation of interconnected phases. Furthermore, the increased mobility contradicts the study of Foster et al., who found a nearly constant hole mobility, but a decreased electron mobility in optimized devices using the space-charge-limited current (SCLC) method^53^. This contradiction might result from the fact that *μ* as derived from OTRACE transients provide an estimate of an effective *μ* including both electron and hole mobility and the influence of trap states^56^. In addition, the 1% DIO device shows the overall highest mobility. Together with an even higher fast photogeneration (Fig. 3d) and a similar field dependence (Supplementary Fig. S5) relative to the 3% DIO blend, this raises the question why the 1% DIO device shows a reduced PCE. It is conceivable that for 1% DIO the mobility is mostly determined by charge carriers extracted from well connected regions representing only a fraction of the bulk and that remaining, less connected domains cause an inefficient charge carrier collection as already suggested in the context of field dependent photogeneration.

Besides charge carrier transport, OTRACE yields information about the density of charge carriers available for extraction under working conditions. Figure 5 shows the charge carrier density *n* as a function of delay time *t_d_* for devices with 0%, 1%, 3%, and 10% DIO. An iterative correction was applied (Supplementary Fig. S6). Considering the shortest *t_d_* of 100 ns, the extracted charge carrier density *n* increases with the addition of DIO by about 50% (1% DIO) and 94% (3% DIO) with respect to the device without additive and again decreases by about 20% (10% DIO) with respect to 3% DIO. The trend is in contrast to the fast photogeneration in Fig. 3d, showing a significant increase in the transient absorption for 1% DIO and a comparable signal height for 3% and 10% DIO. In analogy to the discussion of TA in relation to device performance in the last paragraph, the discrepancy between TA and OTRACE can be explained by two effects. First, enhanced geminate losses during *t_d_* in the case of the 1% and 10% DIO device (Fig. 1b and Supplementary Fig. S5) might result in a reduced charge carrier density in the OTRACE experiment. Second, an extracted charge carrier density, which is smaller, in relative terms, than expected from TA indicates a less efficient polaron pair dissociation in intermixed D-A phases. In addition, an effective extraction requires the adequate collection of charge carriers in interconnected D and A phases. Thus, a fine-tuning of the blend morphology when going from 1% to 3% DIO in terms of a demixing of the matrix phases and the formation of percolation pathways seems reasonable. Similarly, an again reduced charge carrier density for 10% DIO supports the assumption of a reduced linking of pure domains and an incomplete extraction under solar cell working conditions.

The alteration of the blend morphology induced by DIO is also reflected in the time dependent decay of *n*. Figure 5 shows a fast recombination regime at short times and high *n* (hereinafter referred to as *f* ≡ fast), followed by the slow decline of *n(t)* at long times and small *n* (*s* ≡ slow), respectively. Both regimes follow a power law behavior *n(t) ~ t^−1/λ^* yielding a substantial decrease from *λ_f_ = * 3.4 (0% DIO) to 2.5 (1% DIO), a minimum value of 2.3 (3% DIO) and a renewed increase to 3.2 (10% DIO). In contrast, *λ_s_* is about 5.5 and unaffected by the amount of DIO up to 3%. Only the addition of 10% DIO leads to an increase in *λ_s_* to about 7. The deviation of *λ_f_* from pure 2^nd^ order kinetics (*λ +* 1 = 2) indicates a recombination affected by trapped charge carriers^57^,^58^,^59^. Therefore, the presence of both free-free and free-trapped recombination processes are expected over the whole range of *t_d_*. The occurrence of a fast and slow regime can be understood within the scenario of a recombination dominated by spatial trapping at short times and slowed down thermal detrapping at long times. Thus, the maximum value of *λ_f_* in the case of the 0% DIO device agrees well with the scenario of charge carriers spatially trapped inside isolated PC_71_BM devices as suggested by Foertig et al. for PTB7:PC_71_BM^54^. The constant *λ_s_* for 0%, 1%, and 3% DIO can be interpreted as the slope of an exponential density of states (DOS) *E_U_ = λ_s_**·**k_B_T* ≈ 140 meV^60^,^61^, which is independent of the blend morphology. The strong decrease in *λ_f_* already for the small amount of 1% DIO can be seen as a further indication of shrinking acceptor domains, which continues up to a DIO content of 3%. The following increase in *λ_f_* for 10% DIO compared to 3% DIO is consistent with the growth of polymer phases. However, regarding the substantial increase in *λ_s_* and the fairly large *E_U_* of about 180 meV, it cannot be excluded that the over-concentration of DIO strongly influences the energetics of the system in addition to nanostructural changes. One possibility would be a substantially enhanced trap density or the presence of deep tail states due to DIO molecules remaining in the active layer. It must be noted that a possible overestimation of *E_U_*, e.g., as compared to charge extraction measurements on PTB7:PC_71_BM blends^54^, might be explained by the OTRACE technique itself and is a subject of active study. Furthermore, the determination of *λ_s_* might also depend on the film thickness^62^.

## Conclusions

In the present work we report on the use of a systematically increasing fraction of the solvent additive DIO in PBDTTT-C:PC_71_BM solar cells. By combining surface imaging, photophysical and charge extraction techniques we found a multi-tiered effect of DIO on the blend microstructure and correlated changes in photogeneration, free carrier formation and recombination. The measurements reveal a morphology of the blend processed without DIO dominated by large PC_71_BM agglomerates embedded in a PBDTTT-C-rich matrix. The poor photogeneration due to an accordingly small interfacial area and increased singlet exciton losses inside large fullerene domains results in a PCE of 2.6%. It is improved by the use of already small amounts of DIO (0.6%), which leads to a decrease in the size of PC_71_BM domains. Despite the more efficient exciton dissociation due to a strongly enhanced D-A interface, the PCE of the 0.6% DIO device reaches a moderate level of only 5.3%. It is explained by both the photogeneration of predominantly bound polaron pairs and the inefficient collection of free charge carriers in a finely intermixed D-A matrix without a sufficient number of percolation paths. By an increase in the DIO content up to 3% the photogeneration stays on a high level due to the balanced effects of the further shrinking size of PC_71_BM domains and a growth of pure PBDTTT-C regions. Both these factors favor free carrier formation and collection yielding a maximum PCE of 6.9%. It is limited by a dominant nongeminate recombination and by substantially reduced but still present geminate losses. An increase in the DIO content up to 10% results in a reduced PCE of 5.3% mostly due to a decrease in *j_sc_*. Since we found a still highly efficient exciton separation, the reduced performance is presumably caused by a less efficient formation of free charge carriers and their hindered extraction due to less interconnected pure domains. Our study demonstrates that the maximum PCE results from an optimization of the blend microstructure with respect to a crucial balance between local photogeneration and charge carrier transport. It provides a better understanding of the important correlation between the additive content and the increase in solar cell performance.

## Methods

### Sample preparation

All active layers were spin cast from chlorobenzene (CB) solutions under inert atmosphere using a blend of PBDTTT-C (Solarmer Materials Inc.) and PC_71_BM (Solenne) in a 1:1.5 ratio, optionally with 0.6%, 1%, 3%, and 10% by volume of DIO, resulting in a film thickness in the range of 100 to 120 nm. BHJ solar cells were fabricated on indium tin oxide (ITO) covered glass substrates. A 40 nm thin film of PEDOT:PSS (poly(3,4-ethylenedioxythiophene):poly(styrenesulfonate), Clevios Al 4083) was followed by the active layer as described above. In the final step the metal cathode consisting of Ca (3 nm) and Al (120 nm) was evaporated defining the active area of 3 mm^2^. For PL, TA and AFM measurements, sapphire substrates and PEDOT:PSS covered glass substrates were used, respectively. Solar cell characterization was performed under inert atmosphere using a Keithley 237 SMU and an Oriel 1160 AM1.5G solar simulator. It was calibrated to 100 mW cm^−2^.

### AFM

IC-AFM measurements were performed with a NanoWizard ll (JPK Instruments AG, Berlin, Germany) with silicon cantilevers (Pointprobe NCH, NanoWorld AG, Neuchâtel, Switzerland) under ambient conditions. The typical resonance frequency was *ω_0_* ≈ 290 kHz, the free amplitude *A_0_* ≈ 60 nm and the amplitude setpoint *A/A_0_* ≈ 0.9. The RMS roughness of the height images were calculated with the JPK Data Processing software (Version 4.3.52) for (10 × 3.5) μm^2^ large areas.

### TDCF

The device was mounted under inert atmosphere in an closed cycle optical cryostat. Short laser pulses (<80 ps) of a 2.33 eV neodymium doped yttrium aluminum garnet (Nd:YAG) laser with a repetition rate of 5 Hz was used for charge carrier generation. The TDCF pulse consisting of a constant pre-bias voltage *V_pre_* during the delay time *t_d_* followed by the collection voltage *V_coll_* was applied using a pulse function arbitrary noise generator (Agilent 81150A). For all pre-bias dependent measurements a constant delay time of 20 ns, a pulse fluence between 0.3 μJ cm^−2^ and 0.5 μJ cm^−2^ and a pre-bias varying between −4 V and *V_oc_* was used. The resulting current transient was recorded by a digital storage oscilloscope (Agilent Infiniium DSO90254A). For more experimental details, see Reference 41.

### OTRACE

The measurements were performed instantaneously after TDCF measurements using the same digital storage oscilloscope and function generator. The device was illuminated by a high power white light emitting diode. In a first step, a 1.5 GΩ input resistance of a voltage amplifier was used to record the *V_oc_* transient. In a second step the OTRACE pulse, consisting of *V_oc_(t)* during the delay time *t_d_* and a subsequent triangular voltage ramp with a slope of *A = * 90 kV s^−1^, was applied. A more detailed description of the OTRACE technique can be found in the Supplementary Information and in Reference 25.

### PL

For the time-resolved PL measurements, the output of a Ti:Sa oscillator (Spectra Physics Mai Tai, 100 fs, 795 nm) was frequency doubled and focused onto the sample, which was mounted inside a liquid helium cryostat, using a fluence of 12 nJ cm^−2^. The PL was spectrally dispersed by a spectrograph and detected with a streak camera (Hamamatsu C 5680-22). The temporal resolution of the measurements was 18 ps and the detection window limited to 1.5 ns.

### TA

As excitation source for the femtosecond TA measurements the output of a regenerative amplifier system (Spectra Physics Spitfire Pro, 118 fs, 1 mJ, 795 nm) is used to pump two optical parametric amplifiers (OPA, Light Conversion TOPAS-C). The output of the first OPA is employed as excitation source yielding 110 fs pump pulses centered at 480 nm using a fluence of 9.4 μJ cm^−2^ while the second OPA generates probe pulses in the IR spectral range. The pump pulses are delayed with respect to the probe pulses using a motorized linear stage and every second pump pulse is blocked using an optical chopper in order to detect pump-induced absorption changes only. The beam path of the excitation source is stabilized using an automated beam alignment system (TEM Messtechnik Aligna). The transmitted probe pulses are detected with a pre-amplified nitrogen cooled photodiode and the response of the detector for every pulse is integrated using a single channel boxcar before being recorded. The described transient absorption setup has a temporal resolution of around 400 fs as determined by a cross-correlation measurement.

## Author Contributions

The research was planned by C.D., A.Z. and B.G. prepared the samples. *j*-*V* characterization of thin film devices was performed by A.Z., M.Z. measured IC-AFM. B.G. performed PL and TA measurements. A.Z. measured TDCF and OTRACE. A.Z. and B.G. wrote the main article. All authors contributed with discussion, feedback and comments on the manuscript. C.D., R.M. and V.D. supervised the research.

## Supplementary Material

Supplementary Information Supplementary Information
