# Supplementary material for: The Effect of Diiodooctane on the Charge Carrier Generation in Organic Solar Cells Based on the Copolymer PBDTTT-C
Source: Sci Rep. 2015 Feb 6;5:8286. doi: 10.1038/srep08286 (PMC4319164; doi:10.1038/srep08286)
Supplement: Supplementary Information [file srep08286-s1.pdf]

Supplementary Information

The Effect of Diiodooctane on the Charge Carrier Generation in Organic Solar Cells Based on the Copolymer PBDTTT-C

Andreas Zusan,[1,*] Björn Gieseking,[1,*] Mario Zerson,[2] Vladimir Dyakonov,[1,3] Robert Magerle,[2,†] and Carsten Deibel[1,‡]

[1]*Experimental Physics VI, Julius-Maximilians-University of Würzburg, 97074 Würzburg, Germany*

[2]*Fakultät für Naturwissenschaften, Technische Universität Chemnitz, 09126 Chemnitz, Germany*

[3]*Bavarian Centre for Applied Energy Research e.V. (ZAE Bayern), 97074 Würzburg, Germany*

[*] These authors contributed equally to this work.

[†] robert.magerle@physik.tu-chemnitz.de

[‡] deibel@physik.tu-chemnitz.de; Present address: Institute of Physics, Chemnitz University of Technology, 09126 Chemnitz, Germany

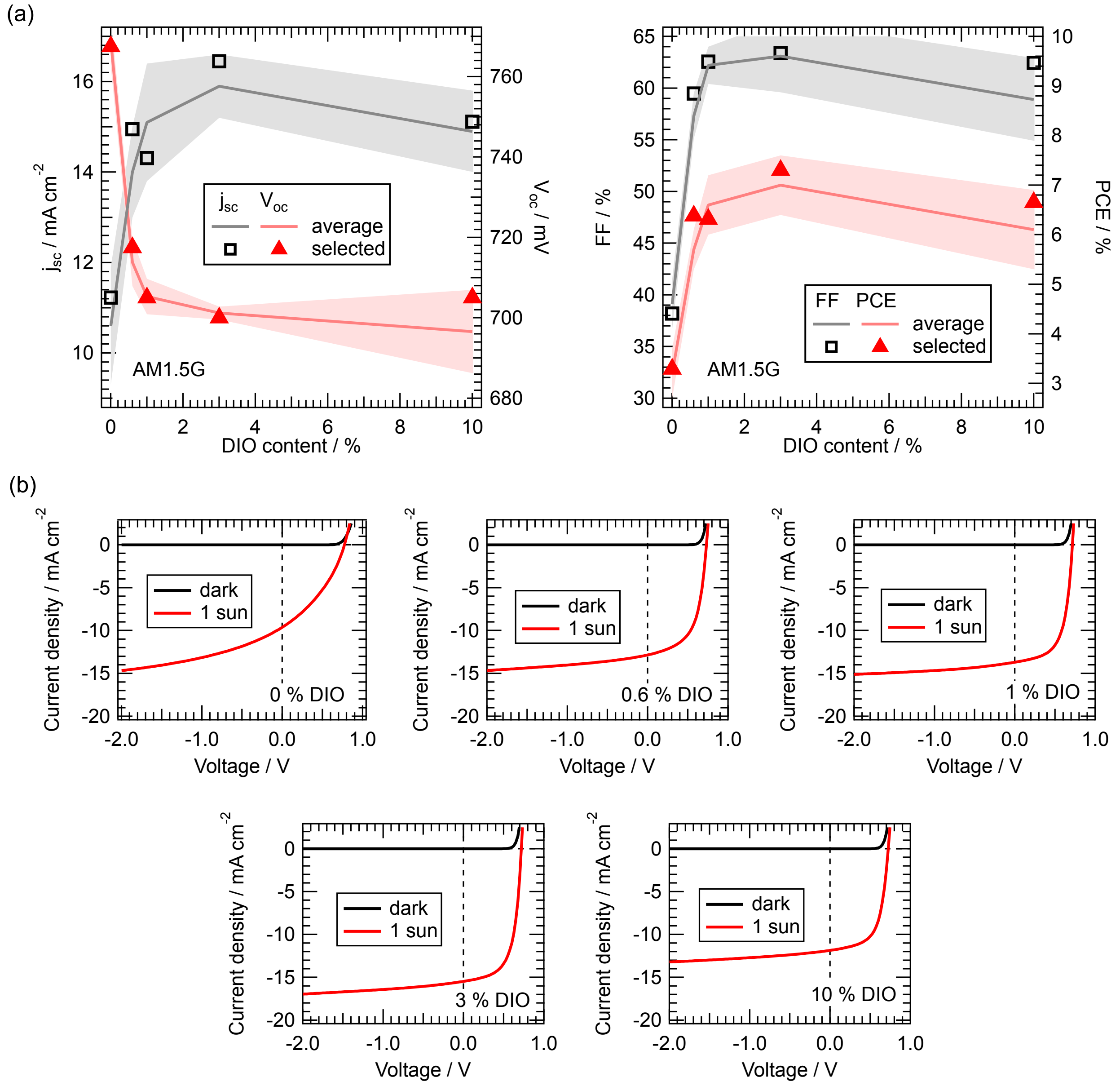

FIG. S1. (a) Photovoltaic parameters of PBDTTT-C:$PC_{71}BM$ devices as a function of the DIO content. The parameters were averaged over a series of 24 (0 % DIO), 14 (0.6 % DIO), 27 (1 % DIO), 17 (3 % DIO), and 16 (10 % DIO) solar cells. The shaded areas illustrate the standard deviation. The symbols correspond to the photovolataic parameters of the devices selected for TDCF and OTRACE measurements. These samples were chosen with respect to device performance and dark saturation current. The shown parameters were measured immediately after sample preparation under a simulated AM1.5G spectrum. (b) *j*-*V* characteristics and dark currents of the selected PBDTTT-C:$PC_{71}BM$ devices measured in the optical cryostat before TDCF and OTRACE experiments. The illumination intensity of the high power white light emitting diode was set to 1 sun. The corresponding photovoltaic parameters can be found in Table S1.

	PBDTTT-C:$PC_{71}BM$				
	0 % DIO	0.6 % DIO	1 % DIO	3 % DIO	10 % DIO
V_{oc} / mV	773	731	715	719	724
j_{sc} / mA cm^{-2}	9.6	12.8	13.7	15.5	11.9
FF / %	35.4	56.9	60.8	61.7	61.1
PCE / %	2.6	5.3	6.0	6.9	5.3

TAB. S1. Photovoltaic parameters of the selected PBDTTT-C:$PC_{71}BM$ solar cells shown in Fig. S1b.

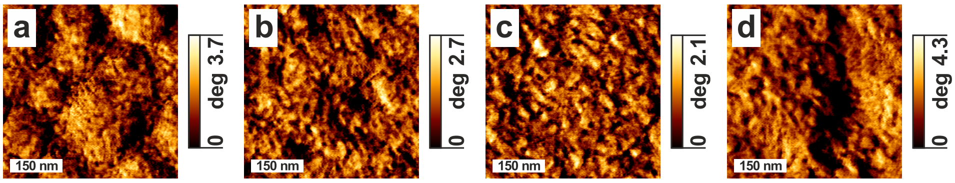

FIG. S2. High-resolution IC-AFM phase images of thin films of PBDTTT-C:$PC_{71}BM$ blends prepared with 0 % (a), 0.6 % (b), 3 % (c) and 10 % (d) of DIO. The panels correspond to the magnified height images shown in the right column of Fig. 2 in the main text.

	time-integrated		transient		
	A_D	A_A	τ_1 / ps	τ_2 / ps	A_1/A_2
$PC_{71}BM$	—	—	—	726	—
PBDTTT-C	—	—	—	280	—
0 % DIO	—	3530	24	512	0.29
1 % DIO	230	2330	28	264	0.97
3 % DIO	690	700	18	145	1.47
10 % DIO	1180	470	14	186	2.80

TAB. S2. Fit amplitudes of donor and acceptor contributions to the integral blend PL as well as time constants and corresponding amplitude ratio derived from the analysis of the time-resolved PL measurements of PBDTTT-C:$PC_{71}BM$ solar cells with varying DIO content. For comparison also the decay times for neat $PC_{71}BM$ and PCDTTT-C films are shown. τ_2 denotes the effective radiative lifetime of fullerene singlet excitons whereas τ_1 can be related to the dissociation of excitons at the D-A interface.

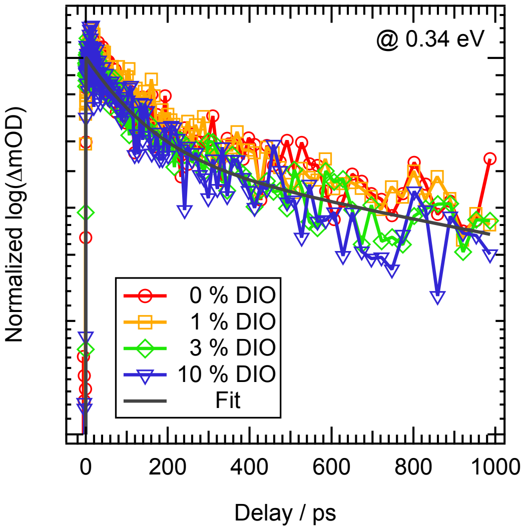

FIG. S3. TA transients probed at 0.34 eV for the different blends with 0 %, 1 %, 3 % and 10 % DIO. Assuming a biexponential decay, the fit yields a fast and a slow time constant of $\tau_1 = 100$ ps and $\tau_2 = 2$ ns, respectively, with an amplitude ratio A_1/A_2 of around 0.8. Considering the ps to ns time regime the probed species can be interpreted as bound charge pairs with τ_1 being in agreement with reported lifetimes for CT excitons.[1] In this context τ_1 might represent a loss channel, e.g. resulting from the diffusion of the opposite charges away from the interface.[2]

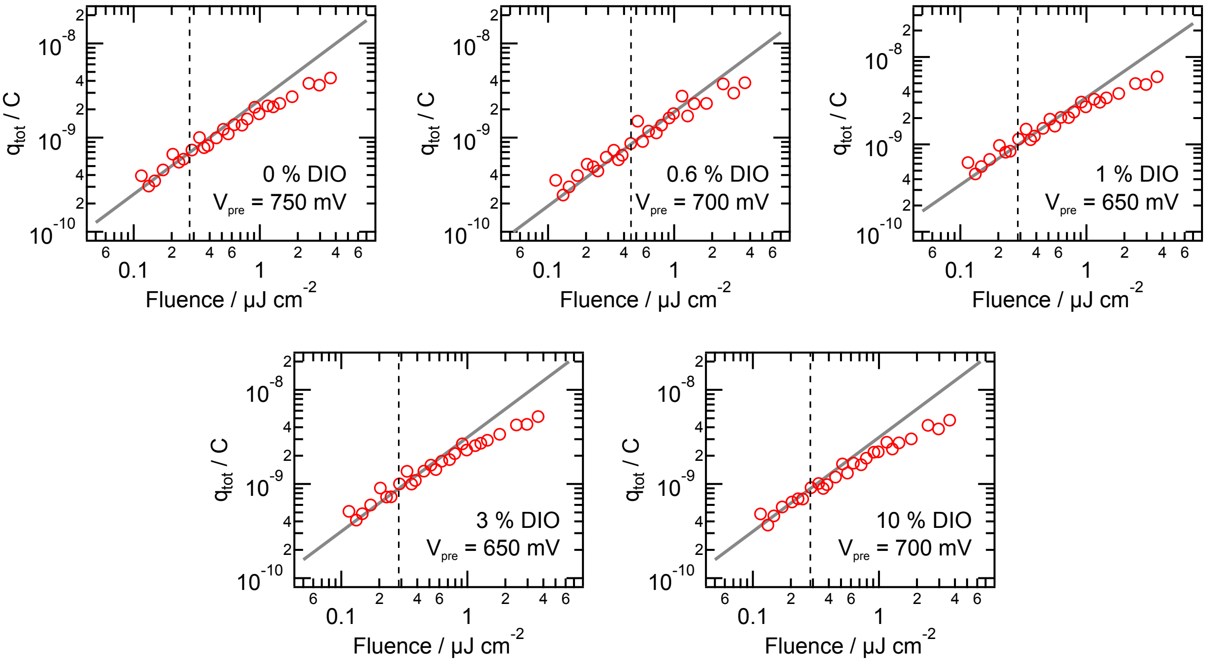

FIG. S4. Integrated charge q_{tot} as a function of the pulse fluence derived from TDCF measurements on PBDTTT-C:PC_{71}BM solar cells. The dashed line indicates the pulse fluence which was chosen for the pre-bias dependent measurements. In order to avoid nongeminate recombination, the laser intensity was adjusted to be in range showing a photogeneration linear proportional to illumination intensity (grey line). The delay time was set to 20 ns and the collection voltage to -4 V for all measurements.

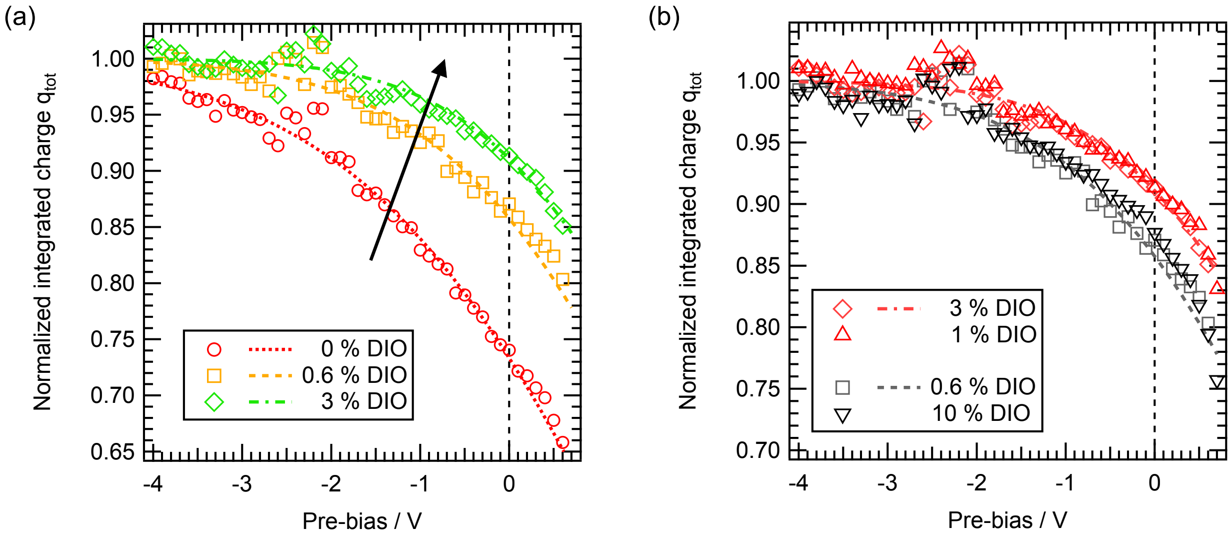

FIG. S5. (a) Integrated charge q_{tot} as a function of pre-bias V_{pre} (open symbols) derived from TDCF measurements on PBDTTT-C:PC_{71}BM solar cells. As guide to the eyes, q_{tot} was fitted by Gaussian (lines) and normalized to the respective maximum of the fit. The black arrow indicates a decreasing field dependence. For the sake of clarity, only data for 0 %, 0.6 % and 3 % DIO are shown. The normalized q_{tot} obtained on the 1 % and 10 % DIO device roughly coincide with data of the 3 % DIO and 0.6 % DIO device, respectively, as it can be found on the right. (b) Integrated charge q_{tot} vs. pre-bias V_{pre} derived from TDCF on PBDTTT-C:PC_{71}BM solar cells with 0.6 %, 1 %, 3 % and 10 % DIO. Data were normalized to the maximum of the respective Gaussian fit (0.6 % and 3 % DIO).

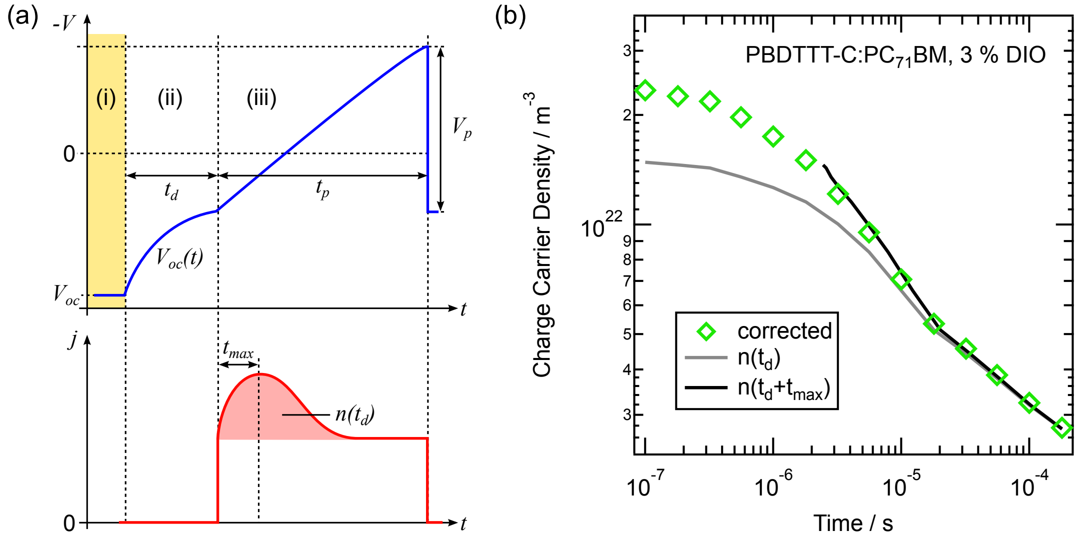

FIG. S6. (a) The applied OTRACE pulse $V(t)$ in reverse direction at a certain delay time t_d and a schematic illustration of the current transient $j_{t_d}(t)$. Three different regimes can be distinguished: (i) charge carrier generation by a high power white light emitting diode, (ii) charge carrier recombination under open circuit conditions and (iii) extraction of the remaining charge carriers by a triangular voltage pulse with the height V_p and the length t_p.[3] To account for recombination losses during extraction,[4, 5] the density of extracted charge carriers $n(t_d)$ was corrected iteratively. A first estimate of the initial charge carrier density at a certain delay time $n_{it0}(t_d)$ was calculated by integrating OTRACE transients $j_{t_d}(t)$ as usual. In the next step, the number of charge carriers remaining within the sample during extraction was calculated from $n_{it0}(t_d)$ and $j_{t_d}(t)$. It was used to compute the corresponding recombination rate R using the recombination coefficient k_λ and order λ, which are extracted from a previous fit of $n_{it0}(t_d)$. The number of recombined charges $n_R(t_d)$ was subsequently determined by integrating $dn/dt = -k_\lambda n^{\lambda+1}$:

$$n_R(t_d) = \int_0^{t_p} k_\lambda \cdot \left(n_{it0}(t_d) - \frac{1}{q \cdot d}\int_0^t j_{t_d}(t')\, dt'\right)^{\lambda+1} dt \ ,$$

with the sample thickness d and the elementary charge q. Finally, $n_R(t_d)$ is added to $n_{it0}(t_d)$ and the calculation was repeated until convergence was found. (b) Comparison of the charge carrier density as function of delay time t_d and as function of the sum of delay time t_d and the maximum peak position t_{max} with the iteratively corrected $n_{corr}(t_d)$. Data were derived from OTRACE measurements under 1 sun illumination intensity on PBDTTT-C:PC$_{71}$BM solar cells processed with 3 % DIO.

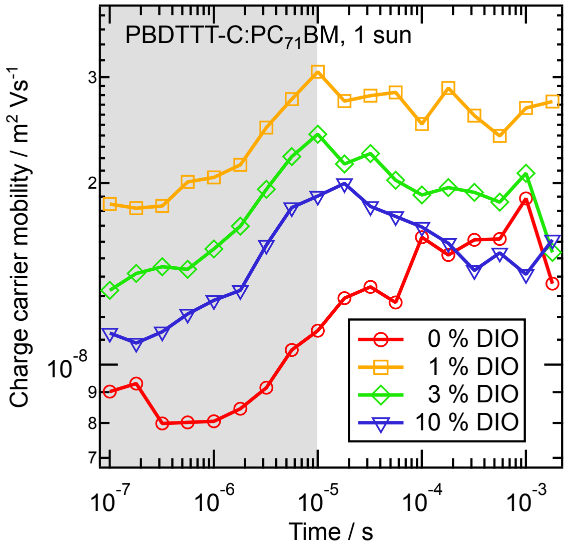

FIG. S7. Charge carrier mobility μ vs. delay time t_d derived from the maximum peak position t_{max} of OTRACE transients according to Ref. [6] for a light intensity of 1 sun. The increase of μ in the shaded area is attributed to RC distortions.[3] Values of $1.47 \pm 0.22 \cdot 10^{-8}$ m^2 (Vs)$^{-1}$, $2.72 \pm 0.19 \cdot 10^{-8}$ m^2 (Vs)$^{-1}$, $2.01 \pm 0.24 \cdot 10^{-8}$ m^2 (Vs)$^{-1}$, and $1.68 \pm 0.20 \cdot 10^{-8}$ m^2 (Vs)$^{-1}$ were derived as average values in the non-shaded time range for PBDTTT-C:$PC_{71}BM$ devices with 0 %, 1 %, 3 %, and 10 %, respectively.

[1] Jarzab, D. *et al.* Low-temperature behaviour of charge transfer excitons in narrow-bandgap polymerbased bulk heterojunctions. *Adv. Energy Mater.* **1**, 604–609 (2011).

[2] Barbour, L.W. , Hegadorn, M. & Asbury, J. B. Watching electrons move in real time. *J. Am. Chem. Soc.* **129**, 15884–15894 (2007).

[3] Baumann, A., Lorrmann, J., Rauh, D., Deibel, C. & Dyakonov, V. A new approach for probing the mobility and lifetime of photogenerated charge carriers in organic solar cells under real operating conditions. *Adv. Mater.* **24**, 4381–4386 (2012).

[4] Shuttle, C. G., O'Regan, B., Ballantyne, A. M., Nelson, J., Bradley, D. D. C. & Durrant, J. R. Bimolecular recombination losses in polythiophene:fullerene solar cells. *Phys. Rev. B* **78**, 113201–113205 (2008).

[5] Foertig, A., Wagenpfahl, A., Gerbich, T., Cheyns, D., Dyakonov, V. & Deibel, C. Nongeminate recombination in planar and bulk heterojunction organic solar cells. *Adv. Energy Mater.* **2**, 1483 (2012).

[6] Lorrmann, J., Badada, B. H., Inganäs, O., Dyakonov, V. & Deibel, C. Charge carrier extraction by linearly increasing voltage: Analytic framework and ambipolar transients. *J. Appl. Phys.* **108**, 113705–8 (2010).
